# [μ_2_-Bis(diphenyl­phosphanyl)methane][μ_3_-bis­(diphenyl­phosphanyl)meth­yl]trichlorido­tetra­gold(I) tetra­hydro­furan disolvate

**DOI:** 10.1107/S1600536810054115

**Published:** 2011-01-08

**Authors:** Huan-Huan Wang, Qian Gao, Yue Cui, Lin Wang, Ya-Bo Xie

**Affiliations:** a College of Environmental and Energy Engineering, Beijing University of Technology, Beijing 100124, People’s Republic of China

## Abstract

The title tetra­nuclear complex, [Au_4_(C_25_H_21_P_2_)Cl_3_(C_25_H_22_P_2_)]·2C_4_H_8_O, features two non-equivalent Ph_2_PCPPh_2_ fragments, one of which represents the ‘complete’ mol­ecule (with two H atoms at the central C atom); each of the two P atoms of this mol­ecule is coordinated by an Au atom [Au—P = 2.2256 (13) and 2.2710 (13) Å], and these two Au atoms form an Au—Au bond [3.2945 (3) Å], thus closing the five-membered Au_2_P_2_C ring. The first of these Au atoms has a terminal chlorido ligand [Au—Cl = 2.2806 (12) Å], whereas the second Au atom forms a covalent bond with the central C atom of the bis­(diphenyl­phosphino)methyl group [Au—C = 2.114 (5) Å]; the latter group in turn coordinates with its P atoms the gold atoms of the Cl–Au–Au–Cl group [Au—P = 2.2356 (13) and 2.2338 (13), Au—Au = 3.3177 (3), Au—Cl = 2.3091 (12) and 2.2950 (13) Å], thus closing the second Au_2_P_2_C ring. The two such rings have different chemical functions, but both exhibit envelope conformations. However, the first (with different substituents at the Au atoms) is non-symmetrical with one of the P atoms in the flap position of the envelope; the other one has a conformation with mirror symmetry, and the gold-substituted C atom is displaced by 0.740 (5) Å from the almost exactly planar (r.m.s. deviation = 0.0038 Å) Au_2_P_2_ group.

## Related literature

For the structures of related gold complexes with bis­(di­phenyl­phosphino)methane ligands, see: Bruce *et al.* (2006 ▶); Feng *et al.* (1997 ▶); Sevillano *et al.* (2007 ▶). 
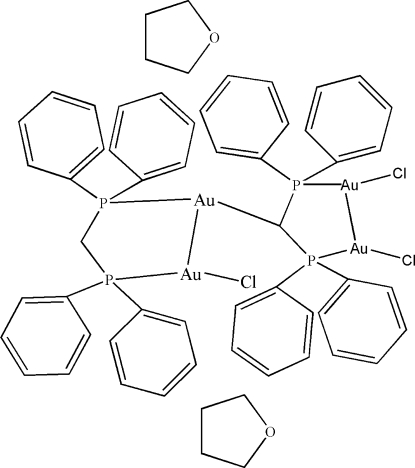


## Experimental

### 

#### Crystal data

[Au_4_(C_25_H_21_P_2_)Cl_3_(C_25_H_22_P_2_)]·2C_4_H_8_O
*M* *_r_* = 1806.15
Monoclinic, 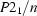

*a* = 14.9822 (6) Å
*b* = 14.6274 (6) Å
*c* = 27.1356 (10) Å
β = 104.981 (1)°
*V* = 5744.7 (4) Å^3^
*Z* = 4
Mo *K*α radiation
μ = 10.47 mm^−1^
*T* = 100 K
0.22 × 0.20 × 0.18 mm

#### Data collection

Bruker SMART CCD area-detector diffractometer
Absorption correction: multi-scan (*SADABS*; Bruker, 1998 ▶) *T*_min_ = 0.115, *T*_max_ = 0.152
31460 measured reflections
10683 independent reflections
9041 reflections with *I* > 2σ(*I*)
*R*_int_ = 0.034

#### Refinement

*R*[*F* ^2^ > 2σ(*F* ^2^)] = 0.027
*wR*(*F* ^2^) = 0.058
*S* = 0.99
10683 reflections
640 parameters
H-atom parameters constrained
Δρ_max_ = 1.32 e Å^−3^
Δρ_min_ = −0.61 e Å^−3^

### 

Data collection: *SMART* (Bruker, 1998 ▶); cell refinement: *SAINT* (Bruker, 1998 ▶); data reduction: *SAINT*; program(s) used to solve structure: *SHELXS97* (Sheldrick, 2008 ▶); program(s) used to refine structure: *SHELXL97* (Sheldrick, 2008 ▶); molecular graphics: *SHELXTL* (Sheldrick, 2008 ▶); software used to prepare material for publication: *SHELXTL*.

## Supplementary Material

Crystal structure: contains datablocks I, global. DOI: 10.1107/S1600536810054115/ya2134sup1.cif

Structure factors: contains datablocks I. DOI: 10.1107/S1600536810054115/ya2134Isup2.hkl

Additional supplementary materials:  crystallographic information; 3D view; checkCIF report

## Figures and Tables

**Fig. 1.**
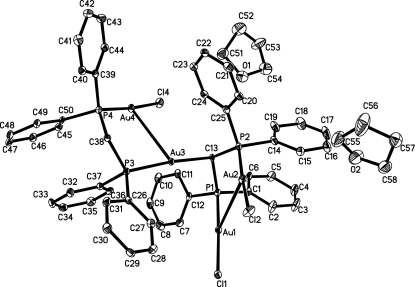
The molecular structure of the title compound with displacement ellipsoids drawn at the 30% probability level; hydrogen atoms are omitted.

## supplementary crystallographic information

### Comment

The crystal structures of Au compounds with the bis(diphenylphosphanyl)methane ligand have been widely studied (see, for instance, Bruce *et al.*, 2006; Feng *et al.*, 1997; Sevillano *et al.*, 2007). Herein, we report the bis(tetrahydrofurane) solvate of a new tetranuclear complex with this ligand and a rather peculiar non-symmetrical structure.

The molecule of the title compound, (Fig. 1) features two chemically different fragments derived from bis(diphenylphosphanyl)methane, one of which represents neutral molecule coordinated to two gold atoms through each of its P atoms [Au3-P3 2.2710 (13), Au4-P4 2.2256 (13) Å]. These gold atoms are bonded to each other [Au3-Au4 3.2945 (3) Å] thus closing the Au_2_P_2_C-ring. The Au4 atom also has a terminal chloro ligand [Au4-Cl4 2.2806 (12) Å], whereas the Au3 atom is bonded to the C13 atom belonging to the bis(diphenylphosphanyl)methyl group [Au3-C13 2.114 (5) Å], which in its turn has each of its phosphorus atoms P1 and P2 bonded to gold atoms of the Cl1-Au1-Au2-Cl2 fragment [Au1-P1 2.2356 (13) Å, Au2-P2 2.2338 (13) Å, Au1-Au2 3.3177 (3) Å, Au1-Cl1 2.3091 (12) Å, Au2-Cl2 2.2950 (13) Å], thus closing the second five-membered ring. Both five-memebered Au_2_P_2_C rings have envelope conformations, however the conformation of symmetrically substituted ring with Au1 and Au2 atoms has a mirror symmetry [displacement of the C13 atom from the almost exactly planar Au_2_P_2_ group being 0.740 (5) Å], whereas the second five-membered ring is not symmetrical with one of the phosphorus atoms (P3) in the flap position, its displacement from the plane of the rest of the atoms in this ring being 0.889 (1) Å.

### Experimental

A solution of AuCl (0.1 mmol, 23.5 mg) in 1 ml of H_2_O and bis(diphenylphosphanyl)methane (0.2 mmol, 77 mg) in 1 ml of THF were mixed and sealed in a 4 ml glass vial and kept at 333 K for 2 days. Then the mixture was cooled down to room temperature and was allowed to stand. After 5 days, colorless crystals suitable for the X-ray diffraction experiment were collected.

### Refinement

All H atoms were placed geometrically (C-H 0.95 Å and 0.99 Å for aromatic and methylene H atoms respectively) and treated as riding with *U*_iso_(H) = 1.2*U*_eq_(C). There are four residual peaks higher than 1 eÅ^-3^, each one in the vicinity of each of the Au atom at the distances of 0.881-0.991 Å.

### Crystal data

**Table:**

[Au_4_(C_25_H_21_P_2_)Cl_3_(C_25_H_22_P_2_)]·2C_4_H_8_O	*F*(000) = 3400
*M**_r_* = 1806.15	*D*_x_ = 2.088 Mg m^−^^3^
Monoclinic, *P*2_1_/*n*	Mo *K*α radiation, λ = 0.71073 Å
Hall symbol: -P 2yn	Cell parameters from 9747 reflections
*a* = 14.9822 (6) Å	θ = 2.3–28.3°
*b* = 14.6274 (6) Å	µ = 10.47 mm^−^^1^
*c* = 27.1356 (10) Å	*T* = 100 K
β = 104.981 (1)°	Block, colorless
*V* = 5744.7 (4) Å^3^	0.22 × 0.20 × 0.18 mm
*Z* = 4	

### Data collection

**Table:**

Bruker SMART CCD area-detector diffractometer	10683 independent reflections
Radiation source: fine-focus sealed tube	9041 reflections with *I* > 2σ(*I*)
graphite	*R*_int_ = 0.034
phi and ω scans	θ_max_ = 25.5°, θ_min_ = 1.4°
Absorption correction: multi-scan (*SADABS*; Bruker, 1998)	*h* = −18→15
*T*_min_ = 0.115, *T*_max_ = 0.152	*k* = −17→16
31460 measured reflections	*l* = −32→32

### Refinement

**Table:**

Refinement on *F*^2^	Primary atom site location: structure-invariant direct methods
Least-squares matrix: full	Secondary atom site location: difference Fourier map
*R*[*F*^2^ > 2σ(*F*^2^)] = 0.027	Hydrogen site location: inferred from neighbouring sites
*wR*(*F*^2^) = 0.058	H-atom parameters constrained
*S* = 0.99	*w* = 1/[σ^2^(*F*_o_^2^) + (0.0278*P*)^2^] where *P* = (*F*_o_^2^ + 2*F*_c_^2^)/3
10683 reflections	(Δ/σ)_max_ = 0.002
640 parameters	Δρ_max_ = 1.32 e Å^−^^3^
0 restraints	Δρ_min_ = −0.61 e Å^−^^3^

### Special details

**Table:**

Geometry. All esds (except the esd in the dihedral angle between two l.s. planes) are estimated using the full covariance matrix. The cell esds are taken into account individually in the estimation of esds in distances, angles and torsion angles; correlations between esds in cell parameters are only used when they are defined by crystal symmetry. An approximate (isotropic) treatment of cell esds is used for estimating esds involving l.s. planes.
Refinement. Refinement of F^2^ against ALL reflections. The weighted R-factor wR and goodness of fit S are based on F^2^, conventional R-factors R are based on F, with F set to zero for negative F^2^. The threshold expression of F^2^ > 2sigma(F^2^) is used only for calculating R-factors(gt) etc. and is not relevant to the choice of reflections for refinement. R-factors based on F^2^ are statistically about twice as large as those based on F, and R- factors based on ALL data will be even larger.

### Fractional atomic coordinates and isotropic or equivalent isotropic displacement parameters (Å^2^)

**Table:**

	*x*	*y*	*z*	*U*_iso_*/*U*_eq_	
Au1	0.821477 (13)	0.165214 (13)	0.399909 (7)	0.01754 (5)	
Au2	0.729665 (13)	0.371899 (13)	0.398530 (7)	0.01955 (5)	
Au3	0.830843 (12)	0.267477 (13)	0.528113 (7)	0.01567 (5)	
Au4	0.821891 (13)	0.276224 (13)	0.648058 (8)	0.01946 (5)	
Cl1	0.92100 (8)	0.18934 (9)	0.34889 (5)	0.0225 (3)	
Cl2	0.80684 (10)	0.44543 (10)	0.34719 (5)	0.0330 (3)	
Cl4	0.70061 (9)	0.17746 (9)	0.63746 (5)	0.0295 (3)	
P1	0.73048 (9)	0.12581 (9)	0.45001 (5)	0.0168 (3)	
P2	0.64608 (9)	0.31387 (9)	0.44833 (5)	0.0175 (3)	
P3	0.97673 (9)	0.31433 (9)	0.56659 (5)	0.0161 (3)	
P4	0.93872 (9)	0.37510 (9)	0.66646 (5)	0.0167 (3)	
O1	0.4371 (3)	0.2321 (3)	0.54344 (19)	0.0568 (13)	
O2	0.4516 (4)	0.4614 (3)	0.26736 (19)	0.0697 (16)	
C1	0.6251 (3)	0.0689 (3)	0.41479 (18)	0.0180 (11)	
C2	0.5989 (4)	0.0764 (4)	0.3627 (2)	0.0346 (15)	
H2A	0.6367	0.1085	0.3454	0.041*	
C3	0.5162 (4)	0.0368 (5)	0.3354 (2)	0.0451 (18)	
H3A	0.4968	0.0439	0.2994	0.054*	
C4	0.4635 (4)	−0.0115 (4)	0.3596 (2)	0.0356 (15)	
H4A	0.4077	−0.0390	0.3406	0.043*	
C5	0.4906 (4)	−0.0209 (4)	0.4121 (2)	0.0335 (14)	
H5A	0.4541	−0.0556	0.4291	0.040*	
C6	0.5703 (4)	0.0200 (4)	0.4393 (2)	0.0277 (13)	
H6A	0.5882	0.0147	0.4754	0.033*	
C7	0.8515 (4)	−0.0167 (4)	0.4873 (2)	0.0282 (13)	
H7A	0.8688	−0.0110	0.4562	0.034*	
C8	0.8916 (4)	−0.0829 (4)	0.5219 (2)	0.0406 (16)	
H8A	0.9359	−0.1231	0.5143	0.049*	
C9	0.8682 (4)	−0.0914 (4)	0.5676 (2)	0.0323 (14)	
H9A	0.8961	−0.1376	0.5912	0.039*	
C10	0.8047 (4)	−0.0332 (4)	0.5790 (2)	0.0304 (13)	
H10A	0.7889	−0.0390	0.6105	0.036*	
C11	0.7632 (4)	0.0346 (3)	0.54457 (19)	0.0228 (12)	
H11A	0.7199	0.0756	0.5527	0.027*	
C12	0.7862 (3)	0.0416 (3)	0.49766 (18)	0.0190 (11)	
C13	0.7005 (3)	0.2186 (3)	0.48621 (18)	0.0150 (10)	
H13A	0.6628	0.1967	0.5094	0.018*	
C14	0.5300 (3)	0.2836 (3)	0.4117 (2)	0.0230 (12)	
C15	0.4999 (4)	0.3097 (4)	0.3611 (2)	0.0353 (15)	
H15A	0.5399	0.3431	0.3456	0.042*	
C16	0.4112 (4)	0.2871 (5)	0.3327 (3)	0.0431 (17)	
H16A	0.3900	0.3058	0.2982	0.052*	
C17	0.3556 (5)	0.2382 (5)	0.3550 (3)	0.0507 (19)	
H17A	0.2950	0.2233	0.3355	0.061*	
C18	0.3839 (4)	0.2096 (4)	0.4047 (3)	0.0422 (17)	
H18A	0.3442	0.1736	0.4191	0.051*	
C19	0.4719 (4)	0.2338 (4)	0.4341 (3)	0.0336 (14)	
H19A	0.4917	0.2164	0.4689	0.040*	
C20	0.6101 (3)	0.4881 (3)	0.4785 (2)	0.0230 (12)	
H20A	0.6003	0.5029	0.4434	0.028*	
C21	0.6037 (4)	0.5561 (4)	0.5126 (2)	0.0282 (13)	
H21A	0.5884	0.6168	0.5009	0.034*	
C22	0.6198 (3)	0.5354 (4)	0.5637 (2)	0.0280 (13)	
H22A	0.6185	0.5824	0.5876	0.034*	
C23	0.6378 (4)	0.4463 (4)	0.5800 (2)	0.0303 (13)	
H23A	0.6470	0.4322	0.6152	0.036*	
C24	0.6427 (3)	0.3773 (4)	0.5459 (2)	0.0233 (12)	
H24A	0.6542	0.3160	0.5574	0.028*	
C25	0.6304 (3)	0.3988 (3)	0.49429 (19)	0.0190 (11)	
C26	1.0386 (3)	0.3562 (3)	0.52206 (19)	0.0171 (11)	
C27	1.0185 (4)	0.3184 (3)	0.47360 (19)	0.0215 (12)	
H27A	0.9694	0.2757	0.4638	0.026*	
C28	1.0685 (4)	0.3417 (4)	0.4394 (2)	0.0246 (12)	
H28A	1.0538	0.3152	0.4063	0.030*	
C29	1.1403 (3)	0.4036 (4)	0.4535 (2)	0.0236 (12)	
H29A	1.1767	0.4183	0.4306	0.028*	
C30	1.1587 (4)	0.4440 (4)	0.5008 (2)	0.0287 (13)	
H30A	1.2064	0.4883	0.5100	0.034*	
C31	1.1088 (4)	0.4211 (4)	0.5351 (2)	0.0271 (13)	
H31A	1.1222	0.4496	0.5677	0.033*	
C32	1.1373 (4)	0.2379 (4)	0.6298 (2)	0.0248 (12)	
H32A	1.1635	0.2973	0.6307	0.030*	
C33	1.1891 (4)	0.1670 (4)	0.6566 (2)	0.0280 (13)	
H33A	1.2502	0.1780	0.6765	0.034*	
C34	1.1521 (4)	0.0800 (4)	0.6544 (2)	0.0280 (13)	
H34A	1.1883	0.0315	0.6725	0.034*	
C35	1.0638 (4)	0.0636 (3)	0.6265 (2)	0.0266 (13)	
H35A	1.0388	0.0037	0.6251	0.032*	
C36	1.0102 (4)	0.1348 (3)	0.59980 (19)	0.0207 (11)	
H36A	0.9486	0.1235	0.5805	0.025*	
C37	1.0472 (3)	0.2224 (3)	0.60168 (19)	0.0186 (11)	
C38	0.9833 (3)	0.4068 (3)	0.61248 (18)	0.0163 (11)	
H38A	1.0485	0.4260	0.6253	0.020*	
H38B	0.9478	0.4598	0.5949	0.020*	
C39	0.9083 (3)	0.4827 (3)	0.69037 (18)	0.0182 (11)	
C40	0.9591 (3)	0.5624 (3)	0.68955 (19)	0.0211 (11)	
H40A	1.0100	0.5619	0.6747	0.025*	
C41	0.9355 (4)	0.6418 (4)	0.7103 (2)	0.0272 (13)	
H41A	0.9708	0.6956	0.7100	0.033*	
C42	0.8605 (4)	0.6442 (4)	0.7316 (2)	0.0303 (13)	
H42A	0.8445	0.6992	0.7458	0.036*	
C43	0.8097 (4)	0.5655 (4)	0.7319 (2)	0.0303 (13)	
H43A	0.7583	0.5666	0.7463	0.036*	
C44	0.8327 (3)	0.4859 (4)	0.71156 (19)	0.0228 (12)	
H44A	0.7970	0.4324	0.7119	0.027*	
C45	1.0375 (4)	0.2417 (4)	0.73054 (19)	0.0224 (12)	
H45A	0.9816	0.2076	0.7215	0.027*	
C46	1.1162 (4)	0.2022 (4)	0.76134 (19)	0.0245 (12)	
H46A	1.1150	0.1412	0.7732	0.029*	
C47	1.1964 (4)	0.2527 (3)	0.77444 (19)	0.0235 (12)	
H47A	1.2511	0.2258	0.7951	0.028*	
C48	1.1986 (4)	0.3414 (4)	0.75816 (19)	0.0243 (12)	
H48A	1.2544	0.3755	0.7680	0.029*	
C49	1.1199 (3)	0.3813 (4)	0.72746 (19)	0.0199 (11)	
H49A	1.1209	0.4429	0.7165	0.024*	
C50	1.0389 (3)	0.3297 (3)	0.71279 (18)	0.0162 (11)	
C51	0.4491 (5)	0.2656 (5)	0.5949 (3)	0.054 (2)	
H51A	0.4890	0.3206	0.6008	0.065*	
H51B	0.4780	0.2182	0.6200	0.065*	
C52	0.3534 (5)	0.2887 (6)	0.5999 (3)	0.070 (3)	
H52A	0.3490	0.2831	0.6356	0.084*	
H52B	0.3338	0.3508	0.5870	0.084*	
C53	0.2995 (6)	0.2156 (7)	0.5663 (3)	0.077 (3)	
H53A	0.3036	0.1570	0.5851	0.093*	
H53B	0.2337	0.2332	0.5540	0.093*	
C54	0.3414 (5)	0.2074 (5)	0.5243 (3)	0.058 (2)	
H54A	0.3360	0.1439	0.5112	0.069*	
H54B	0.3106	0.2488	0.4961	0.069*	
C55	0.4912 (7)	0.5337 (6)	0.3012 (3)	0.080 (3)	
H55A	0.5592	0.5338	0.3070	0.096*	
H55B	0.4766	0.5255	0.3344	0.096*	
C56	0.4515 (8)	0.6222 (6)	0.2772 (3)	0.112 (4)	
H56A	0.5011	0.6650	0.2744	0.134*	
H56B	0.4128	0.6515	0.2972	0.134*	
C57	0.3939 (5)	0.5940 (5)	0.2249 (3)	0.0517 (18)	
H57A	0.3272	0.5945	0.2235	0.062*	
H57B	0.4049	0.6350	0.1981	0.062*	
C58	0.4262 (6)	0.5006 (5)	0.2190 (3)	0.058 (2)	
H58A	0.3763	0.4645	0.1963	0.070*	
H58B	0.4797	0.5023	0.2040	0.070*	

### Atomic displacement parameters (Å^2^)

**Table:**

	*U*^11^	*U*^22^	*U*^33^	*U*^12^	*U*^13^	*U*^23^
Au1	0.01512 (11)	0.01976 (11)	0.01662 (11)	−0.00144 (8)	0.00206 (8)	−0.00055 (8)
Au2	0.02057 (11)	0.01999 (11)	0.01713 (11)	−0.00422 (8)	0.00314 (9)	−0.00074 (8)
Au3	0.01249 (10)	0.01809 (10)	0.01494 (11)	−0.00262 (7)	0.00085 (8)	−0.00149 (7)
Au4	0.01640 (11)	0.02231 (11)	0.01836 (11)	−0.00511 (8)	0.00215 (9)	0.00039 (8)
Cl1	0.0166 (7)	0.0295 (7)	0.0206 (7)	0.0000 (5)	0.0035 (5)	0.0009 (5)
Cl2	0.0441 (9)	0.0331 (8)	0.0253 (8)	−0.0208 (6)	0.0152 (7)	−0.0065 (6)
Cl4	0.0243 (7)	0.0347 (8)	0.0292 (8)	−0.0154 (6)	0.0063 (6)	−0.0004 (6)
P1	0.0166 (7)	0.0174 (7)	0.0146 (7)	−0.0021 (5)	0.0011 (6)	−0.0001 (5)
P2	0.0131 (7)	0.0199 (7)	0.0173 (7)	−0.0013 (5)	−0.0003 (6)	−0.0008 (5)
P3	0.0141 (7)	0.0179 (7)	0.0154 (7)	−0.0029 (5)	0.0022 (6)	−0.0009 (5)
P4	0.0156 (7)	0.0178 (7)	0.0151 (7)	−0.0014 (5)	0.0010 (6)	0.0008 (5)
O1	0.053 (3)	0.062 (3)	0.057 (3)	0.003 (2)	0.017 (3)	−0.002 (3)
O2	0.115 (5)	0.045 (3)	0.049 (3)	0.027 (3)	0.019 (3)	0.008 (3)
C1	0.020 (3)	0.015 (3)	0.016 (3)	−0.003 (2)	0.000 (2)	−0.001 (2)
C2	0.034 (4)	0.048 (4)	0.019 (3)	−0.026 (3)	0.002 (3)	0.006 (3)
C3	0.049 (4)	0.063 (5)	0.017 (3)	−0.029 (3)	−0.003 (3)	0.000 (3)
C4	0.027 (3)	0.046 (4)	0.028 (3)	−0.023 (3)	−0.003 (3)	−0.001 (3)
C5	0.033 (4)	0.039 (4)	0.029 (3)	−0.018 (3)	0.010 (3)	0.001 (3)
C6	0.026 (3)	0.037 (3)	0.016 (3)	−0.011 (3)	−0.002 (2)	−0.001 (2)
C7	0.034 (3)	0.027 (3)	0.022 (3)	0.007 (2)	0.004 (3)	−0.001 (2)
C8	0.039 (4)	0.037 (4)	0.041 (4)	0.019 (3)	0.002 (3)	0.002 (3)
C9	0.033 (4)	0.023 (3)	0.032 (3)	0.002 (3)	−0.008 (3)	0.010 (3)
C10	0.034 (3)	0.027 (3)	0.026 (3)	−0.005 (3)	0.000 (3)	0.005 (2)
C11	0.021 (3)	0.019 (3)	0.023 (3)	−0.004 (2)	−0.002 (2)	0.000 (2)
C12	0.019 (3)	0.020 (3)	0.014 (3)	−0.004 (2)	−0.002 (2)	0.001 (2)
C13	0.008 (2)	0.022 (3)	0.013 (3)	−0.0027 (19)	0.001 (2)	0.000 (2)
C14	0.017 (3)	0.018 (3)	0.031 (3)	0.003 (2)	0.000 (2)	−0.002 (2)
C15	0.026 (3)	0.048 (4)	0.027 (3)	0.006 (3)	0.000 (3)	−0.010 (3)
C16	0.023 (3)	0.058 (5)	0.041 (4)	0.005 (3)	−0.005 (3)	−0.010 (3)
C17	0.028 (4)	0.042 (4)	0.065 (5)	0.003 (3)	−0.020 (4)	−0.010 (4)
C18	0.027 (4)	0.033 (4)	0.062 (5)	−0.012 (3)	0.002 (3)	0.004 (3)
C19	0.019 (3)	0.025 (3)	0.051 (4)	0.000 (2)	0.000 (3)	0.000 (3)
C20	0.017 (3)	0.023 (3)	0.029 (3)	0.000 (2)	0.007 (2)	0.000 (2)
C21	0.021 (3)	0.024 (3)	0.041 (4)	−0.002 (2)	0.010 (3)	0.000 (3)
C22	0.014 (3)	0.034 (3)	0.034 (3)	−0.002 (2)	0.003 (3)	−0.014 (3)
C23	0.022 (3)	0.045 (4)	0.023 (3)	0.007 (3)	0.004 (3)	−0.006 (3)
C24	0.017 (3)	0.026 (3)	0.026 (3)	0.003 (2)	0.004 (2)	−0.002 (2)
C25	0.011 (3)	0.022 (3)	0.023 (3)	−0.006 (2)	0.003 (2)	−0.003 (2)
C26	0.017 (3)	0.016 (3)	0.017 (3)	0.005 (2)	0.002 (2)	0.004 (2)
C27	0.022 (3)	0.020 (3)	0.023 (3)	−0.004 (2)	0.005 (2)	0.003 (2)
C28	0.029 (3)	0.024 (3)	0.020 (3)	−0.002 (2)	0.005 (3)	−0.004 (2)
C29	0.017 (3)	0.030 (3)	0.025 (3)	0.005 (2)	0.007 (2)	0.006 (2)
C30	0.022 (3)	0.034 (3)	0.030 (3)	−0.009 (2)	0.006 (3)	−0.005 (3)
C31	0.019 (3)	0.040 (3)	0.021 (3)	−0.011 (2)	0.003 (2)	−0.007 (3)
C32	0.020 (3)	0.026 (3)	0.026 (3)	−0.004 (2)	0.001 (2)	−0.006 (2)
C33	0.015 (3)	0.034 (3)	0.030 (3)	0.005 (2)	−0.003 (3)	−0.003 (3)
C34	0.032 (3)	0.027 (3)	0.025 (3)	0.011 (2)	0.008 (3)	0.003 (2)
C35	0.037 (4)	0.014 (3)	0.029 (3)	0.000 (2)	0.008 (3)	0.002 (2)
C36	0.024 (3)	0.019 (3)	0.019 (3)	−0.002 (2)	0.005 (2)	−0.003 (2)
C37	0.017 (3)	0.020 (3)	0.017 (3)	0.002 (2)	0.001 (2)	−0.002 (2)
C38	0.015 (3)	0.016 (3)	0.018 (3)	−0.002 (2)	0.004 (2)	−0.002 (2)
C39	0.019 (3)	0.019 (3)	0.015 (3)	0.004 (2)	0.001 (2)	0.002 (2)
C40	0.021 (3)	0.023 (3)	0.019 (3)	0.002 (2)	0.004 (2)	0.003 (2)
C41	0.027 (3)	0.019 (3)	0.033 (3)	−0.004 (2)	0.005 (3)	0.001 (2)
C42	0.030 (3)	0.032 (3)	0.029 (3)	0.007 (3)	0.008 (3)	−0.005 (3)
C43	0.031 (3)	0.036 (4)	0.025 (3)	0.006 (3)	0.009 (3)	0.000 (3)
C44	0.018 (3)	0.029 (3)	0.021 (3)	−0.002 (2)	0.005 (2)	−0.001 (2)
C45	0.025 (3)	0.026 (3)	0.016 (3)	−0.004 (2)	0.007 (2)	−0.001 (2)
C46	0.030 (3)	0.024 (3)	0.017 (3)	0.005 (2)	0.001 (2)	0.005 (2)
C47	0.027 (3)	0.024 (3)	0.015 (3)	0.005 (2)	−0.002 (2)	−0.001 (2)
C48	0.020 (3)	0.031 (3)	0.018 (3)	−0.005 (2)	−0.001 (2)	−0.003 (2)
C49	0.019 (3)	0.022 (3)	0.016 (3)	−0.005 (2)	0.001 (2)	−0.001 (2)
C50	0.017 (3)	0.021 (3)	0.009 (2)	−0.001 (2)	0.000 (2)	0.000 (2)
C51	0.050 (5)	0.074 (6)	0.043 (4)	−0.007 (4)	0.017 (4)	−0.006 (4)
C52	0.051 (5)	0.098 (7)	0.073 (6)	−0.031 (4)	0.037 (5)	−0.020 (5)
C53	0.077 (6)	0.105 (8)	0.061 (6)	−0.028 (5)	0.036 (5)	−0.040 (5)
C54	0.048 (5)	0.053 (5)	0.075 (6)	−0.017 (4)	0.022 (4)	−0.008 (4)
C55	0.115 (8)	0.070 (6)	0.045 (5)	0.032 (5)	0.002 (5)	0.000 (4)
C56	0.195 (12)	0.051 (6)	0.057 (6)	0.022 (6)	−0.025 (7)	0.005 (4)
C57	0.062 (5)	0.041 (4)	0.050 (5)	0.001 (3)	0.011 (4)	0.009 (3)
C58	0.078 (6)	0.055 (5)	0.043 (5)	0.014 (4)	0.019 (4)	0.003 (4)

### Geometric parameters (Å, °)

**Table:**

Au1—P1	2.2356 (13)	C24—H24A	0.9500
Au1—Cl1	2.3091 (12)	C26—C27	1.386 (7)
Au1—Au2	3.3177 (3)	C26—C31	1.393 (7)
Au2—P2	2.2338 (13)	C27—C28	1.378 (7)
Au2—Cl2	2.2950 (13)	C27—H27A	0.9500
Au3—C13	2.114 (5)	C28—C29	1.382 (7)
Au3—P3	2.2710 (13)	C28—H28A	0.9500
Au3—Au4	3.2945 (3)	C29—C30	1.375 (7)
Au4—P4	2.2256 (13)	C29—H29A	0.9500
Au4—Cl4	2.2806 (12)	C30—C31	1.377 (7)
P1—C13	1.800 (5)	C30—H30A	0.9500
P1—C1	1.822 (5)	C31—H31A	0.9500
P1—C12	1.825 (5)	C32—C33	1.384 (7)
P2—C13	1.797 (5)	C32—C37	1.387 (7)
P2—C25	1.818 (5)	C32—H32A	0.9500
P2—C14	1.820 (5)	C33—C34	1.382 (8)
P3—C26	1.809 (5)	C33—H33A	0.9500
P3—C37	1.819 (5)	C34—C35	1.366 (7)
P3—C38	1.823 (5)	C34—H34A	0.9500
P4—C39	1.805 (5)	C35—C36	1.397 (7)
P4—C50	1.816 (5)	C35—H35A	0.9500
P4—C38	1.820 (5)	C36—C37	1.393 (7)
O1—C54	1.439 (8)	C36—H36A	0.9500
O1—C51	1.447 (8)	C38—H38A	0.9900
O2—C58	1.391 (8)	C38—H38B	0.9900
O2—C55	1.424 (9)	C39—C40	1.396 (7)
C1—C2	1.369 (7)	C39—C44	1.397 (7)
C1—C6	1.381 (7)	C40—C41	1.376 (7)
C2—C3	1.394 (8)	C40—H40A	0.9500
C2—H2A	0.9500	C41—C42	1.391 (8)
C3—C4	1.351 (8)	C41—H41A	0.9500
C3—H3A	0.9500	C42—C43	1.381 (8)
C4—C5	1.382 (8)	C42—H42A	0.9500
C4—H4A	0.9500	C43—C44	1.371 (7)
C5—C6	1.369 (7)	C43—H43A	0.9500
C5—H5A	0.9500	C44—H44A	0.9500
C6—H6A	0.9500	C45—C50	1.377 (7)
C7—C8	1.374 (8)	C45—C46	1.384 (7)
C7—C12	1.379 (7)	C45—H45A	0.9500
C7—H7A	0.9500	C46—C47	1.377 (7)
C8—C9	1.379 (8)	C46—H46A	0.9500
C8—H8A	0.9500	C47—C48	1.373 (7)
C9—C10	1.370 (8)	C47—H47A	0.9500
C9—H9A	0.9500	C48—C49	1.385 (7)
C10—C11	1.393 (7)	C48—H48A	0.9500
C10—H10A	0.9500	C49—C50	1.396 (7)
C11—C12	1.405 (7)	C49—H49A	0.9500
C11—H11A	0.9500	C51—C52	1.513 (9)
C13—H13A	1.0000	C51—H51A	0.9900
C14—C15	1.385 (8)	C51—H51B	0.9900
C14—C19	1.389 (7)	C52—C53	1.499 (10)
C15—C16	1.392 (8)	C52—H52A	0.9900
C15—H15A	0.9500	C52—H52B	0.9900
C16—C17	1.352 (9)	C53—C54	1.443 (9)
C16—H16A	0.9500	C53—H53A	0.9900
C17—C18	1.371 (9)	C53—H53B	0.9900
C17—H17A	0.9500	C54—H54A	0.9900
C18—C19	1.399 (8)	C54—H54B	0.9900
C18—H18A	0.9500	C55—C56	1.501 (11)
C19—H19A	0.9500	C55—H55A	0.9900
C20—C21	1.378 (7)	C55—H55B	0.9900
C20—C25	1.384 (7)	C56—C57	1.514 (10)
C20—H20A	0.9500	C56—H56A	0.9900
C21—C22	1.378 (8)	C56—H56B	0.9900
C21—H21A	0.9500	C57—C58	1.472 (9)
C22—C23	1.380 (8)	C57—H57A	0.9900
C22—H22A	0.9500	C57—H57B	0.9900
C23—C24	1.385 (7)	C58—H58A	0.9900
C23—H23A	0.9500	C58—H58B	0.9900
C24—C25	1.401 (7)		
			
P1—Au1—Cl1	173.66 (5)	C26—C27—H27A	119.4
P1—Au1—Au2	85.73 (3)	C27—C28—C29	119.6 (5)
Cl1—Au1—Au2	100.61 (3)	C27—C28—H28A	120.2
P2—Au2—Cl2	173.98 (5)	C29—C28—H28A	120.2
P2—Au2—Au1	86.70 (3)	C30—C29—C28	119.6 (5)
Cl2—Au2—Au1	99.29 (4)	C30—C29—H29A	120.2
C13—Au3—P3	174.14 (13)	C28—C29—H29A	120.2
C13—Au3—Au4	106.05 (13)	C29—C30—C31	121.0 (5)
P3—Au3—Au4	79.29 (3)	C29—C30—H30A	119.5
P4—Au4—Cl4	174.11 (5)	C31—C30—H30A	119.5
P4—Au4—Au3	90.94 (3)	C30—C31—C26	120.0 (5)
Cl4—Au4—Au3	94.90 (3)	C30—C31—H31A	120.0
C13—P1—C1	109.0 (2)	C26—C31—H31A	120.0
C13—P1—C12	104.8 (2)	C33—C32—C37	120.0 (5)
C1—P1—C12	103.8 (2)	C33—C32—H32A	120.0
C13—P1—Au1	114.53 (16)	C37—C32—H32A	120.0
C1—P1—Au1	112.38 (16)	C34—C33—C32	120.3 (5)
C12—P1—Au1	111.48 (17)	C34—C33—H33A	119.9
C13—P2—C25	104.9 (2)	C32—C33—H33A	119.9
C13—P2—C14	110.6 (2)	C35—C34—C33	120.3 (5)
C25—P2—C14	105.4 (2)	C35—C34—H34A	119.8
C13—P2—Au2	113.49 (16)	C33—C34—H34A	119.8
C25—P2—Au2	110.31 (16)	C34—C35—C36	120.1 (5)
C14—P2—Au2	111.61 (18)	C34—C35—H35A	120.0
C26—P3—C37	105.7 (2)	C36—C35—H35A	120.0
C26—P3—C38	105.0 (2)	C37—C36—C35	119.8 (5)
C37—P3—C38	105.6 (2)	C37—C36—H36A	120.1
C26—P3—Au3	113.18 (17)	C35—C36—H36A	120.1
C37—P3—Au3	112.17 (17)	C32—C37—C36	119.5 (5)
C38—P3—Au3	114.40 (16)	C32—C37—P3	121.2 (4)
C39—P4—C50	108.1 (2)	C36—C37—P3	119.3 (4)
C39—P4—C38	104.3 (2)	P4—C38—P3	113.3 (3)
C50—P4—C38	104.0 (2)	P4—C38—H38A	108.9
C39—P4—Au4	112.97 (17)	P3—C38—H38A	108.9
C50—P4—Au4	112.27 (16)	P4—C38—H38B	108.9
C38—P4—Au4	114.55 (16)	P3—C38—H38B	108.9
C54—O1—C51	107.4 (5)	H38A—C38—H38B	107.7
C58—O2—C55	105.6 (6)	C40—C39—C44	118.8 (5)
C2—C1—C6	119.4 (5)	C40—C39—P4	122.2 (4)
C2—C1—P1	118.9 (4)	C44—C39—P4	119.0 (4)
C6—C1—P1	121.7 (4)	C41—C40—C39	119.9 (5)
C1—C2—C3	119.6 (5)	C41—C40—H40A	120.0
C1—C2—H2A	120.2	C39—C40—H40A	120.0
C3—C2—H2A	120.2	C40—C41—C42	120.9 (5)
C4—C3—C2	120.6 (6)	C40—C41—H41A	119.6
C4—C3—H3A	119.7	C42—C41—H41A	119.6
C2—C3—H3A	119.7	C43—C42—C41	119.1 (5)
C3—C4—C5	120.0 (5)	C43—C42—H42A	120.4
C3—C4—H4A	120.0	C41—C42—H42A	120.4
C5—C4—H4A	120.0	C44—C43—C42	120.6 (5)
C6—C5—C4	119.7 (5)	C44—C43—H43A	119.7
C6—C5—H5A	120.1	C42—C43—H43A	119.7
C4—C5—H5A	120.1	C43—C44—C39	120.7 (5)
C5—C6—C1	120.7 (5)	C43—C44—H44A	119.7
C5—C6—H6A	119.7	C39—C44—H44A	119.7
C1—C6—H6A	119.7	C50—C45—C46	120.9 (5)
C8—C7—C12	120.3 (5)	C50—C45—H45A	119.5
C8—C7—H7A	119.9	C46—C45—H45A	119.5
C12—C7—H7A	119.9	C47—C46—C45	118.9 (5)
C7—C8—C9	120.7 (6)	C47—C46—H46A	120.6
C7—C8—H8A	119.7	C45—C46—H46A	120.6
C9—C8—H8A	119.7	C48—C47—C46	121.0 (5)
C10—C9—C8	119.9 (5)	C48—C47—H47A	119.5
C10—C9—H9A	120.1	C46—C47—H47A	119.5
C8—C9—H9A	120.1	C47—C48—C49	120.3 (5)
C9—C10—C11	120.5 (5)	C47—C48—H48A	119.9
C9—C10—H10A	119.7	C49—C48—H48A	119.9
C11—C10—H10A	119.7	C48—C49—C50	119.1 (5)
C10—C11—C12	119.1 (5)	C48—C49—H49A	120.4
C10—C11—H11A	120.4	C50—C49—H49A	120.4
C12—C11—H11A	120.4	C45—C50—C49	119.7 (5)
C7—C12—C11	119.5 (5)	C45—C50—P4	120.0 (4)
C7—C12—P1	119.4 (4)	C49—C50—P4	120.1 (4)
C11—C12—P1	121.1 (4)	O1—C51—C52	106.1 (6)
P2—C13—P1	114.4 (3)	O1—C51—H51A	110.5
P2—C13—Au3	105.4 (2)	C52—C51—H51A	110.5
P1—C13—Au3	102.8 (2)	O1—C51—H51B	110.5
P2—C13—H13A	111.3	C52—C51—H51B	110.5
P1—C13—H13A	111.3	H51A—C51—H51B	108.7
Au3—C13—H13A	111.3	C53—C52—C51	99.2 (7)
C15—C14—C19	119.8 (5)	C53—C52—H52A	111.9
C15—C14—P2	119.7 (4)	C51—C52—H52A	111.9
C19—C14—P2	120.5 (4)	C53—C52—H52B	111.9
C14—C15—C16	120.2 (6)	C51—C52—H52B	111.9
C14—C15—H15A	119.9	H52A—C52—H52B	109.6
C16—C15—H15A	119.9	C54—C53—C52	105.3 (6)
C17—C16—C15	119.3 (7)	C54—C53—H53A	110.7
C17—C16—H16A	120.3	C52—C53—H53A	110.7
C15—C16—H16A	120.3	C54—C53—H53B	110.7
C16—C17—C18	122.1 (6)	C52—C53—H53B	110.7
C16—C17—H17A	119.0	H53A—C53—H53B	108.8
C18—C17—H17A	119.0	O1—C54—C53	106.8 (6)
C17—C18—C19	119.3 (6)	O1—C54—H54A	110.4
C17—C18—H18A	120.3	C53—C54—H54A	110.4
C19—C18—H18A	120.3	O1—C54—H54B	110.4
C14—C19—C18	119.3 (6)	C53—C54—H54B	110.4
C14—C19—H19A	120.3	H54A—C54—H54B	108.6
C18—C19—H19A	120.3	O2—C55—C56	108.0 (7)
C21—C20—C25	121.5 (5)	O2—C55—H55A	110.1
C21—C20—H20A	119.3	C56—C55—H55A	110.1
C25—C20—H20A	119.3	O2—C55—H55B	110.1
C20—C21—C22	119.4 (5)	C56—C55—H55B	110.1
C20—C21—H21A	120.3	H55A—C55—H55B	108.4
C22—C21—H21A	120.3	C55—C56—C57	103.7 (7)
C21—C22—C23	120.0 (5)	C55—C56—H56A	111.0
C21—C22—H22A	120.0	C57—C56—H56A	111.0
C23—C22—H22A	120.0	C55—C56—H56B	111.0
C22—C23—C24	120.8 (5)	C57—C56—H56B	111.0
C22—C23—H23A	119.6	H56A—C56—H56B	109.0
C24—C23—H23A	119.6	C58—C57—C56	103.3 (6)
C23—C24—C25	119.2 (5)	C58—C57—H57A	111.1
C23—C24—H24A	120.4	C56—C57—H57A	111.1
C25—C24—H24A	120.4	C58—C57—H57B	111.1
C20—C25—C24	118.9 (5)	C56—C57—H57B	111.1
C20—C25—P2	119.3 (4)	H57A—C57—H57B	109.1
C24—C25—P2	121.7 (4)	O2—C58—C57	107.3 (6)
C27—C26—C31	118.6 (5)	O2—C58—H58A	110.3
C27—C26—P3	118.4 (4)	C57—C58—H58A	110.3
C31—C26—P3	123.0 (4)	O2—C58—H58B	110.3
C28—C27—C26	121.2 (5)	C57—C58—H58B	110.3
C28—C27—H27A	119.4	H58A—C58—H58B	108.5
			
P1—Au1—Au2—P2	−0.37 (5)	C21—C20—C25—P2	−175.6 (4)
Cl1—Au1—Au2—P2	179.53 (5)	C23—C24—C25—C20	−2.6 (7)
P1—Au1—Au2—Cl2	−179.83 (5)	C23—C24—C25—P2	174.4 (4)
Cl1—Au1—Au2—Cl2	0.08 (5)	C13—P2—C25—C20	165.0 (4)
C13—Au3—Au4—P4	−159.40 (14)	C14—P2—C25—C20	−78.2 (4)
P3—Au3—Au4—P4	23.07 (5)	Au2—P2—C25—C20	42.5 (4)
C13—Au3—Au4—Cl4	19.84 (14)	C13—P2—C25—C24	−11.9 (5)
P3—Au3—Au4—Cl4	−157.70 (5)	C14—P2—C25—C24	104.9 (4)
Au2—Au1—P1—C13	−26.52 (17)	Au2—P2—C25—C24	−134.5 (4)
Au2—Au1—P1—C1	98.65 (17)	C37—P3—C26—C27	91.9 (4)
Au2—Au1—P1—C12	−145.32 (17)	C38—P3—C26—C27	−156.7 (4)
Au1—Au2—P2—C13	27.05 (17)	Au3—P3—C26—C27	−31.3 (4)
Au1—Au2—P2—C25	144.44 (18)	C37—P3—C26—C31	−84.7 (5)
Au1—Au2—P2—C14	−98.71 (18)	C38—P3—C26—C31	26.7 (5)
Au4—Au3—P3—C26	−166.56 (17)	Au3—P3—C26—C31	152.1 (4)
Au4—Au3—P3—C37	73.93 (17)	C31—C26—C27—C28	2.3 (8)
Au4—Au3—P3—C38	−46.32 (17)	P3—C26—C27—C28	−174.5 (4)
Au3—Au4—P4—C39	120.06 (17)	C26—C27—C28—C29	0.1 (8)
Au3—Au4—P4—C50	−117.43 (17)	C27—C28—C29—C30	−2.5 (8)
Au3—Au4—P4—C38	0.83 (18)	C28—C29—C30—C31	2.4 (8)
C13—P1—C1—C2	111.2 (5)	C29—C30—C31—C26	0.1 (9)
C12—P1—C1—C2	−137.5 (5)	C27—C26—C31—C30	−2.4 (8)
Au1—P1—C1—C2	−17.0 (5)	P3—C26—C31—C30	174.2 (4)
C13—P1—C1—C6	−67.5 (5)	C37—C32—C33—C34	1.5 (8)
C12—P1—C1—C6	43.8 (5)	C32—C33—C34—C35	−0.8 (8)
Au1—P1—C1—C6	164.4 (4)	C33—C34—C35—C36	−0.1 (8)
C6—C1—C2—C3	1.6 (9)	C34—C35—C36—C37	0.3 (8)
P1—C1—C2—C3	−177.0 (5)	C33—C32—C37—C36	−1.2 (8)
C1—C2—C3—C4	−2.3 (11)	C33—C32—C37—P3	179.3 (4)
C2—C3—C4—C5	1.0 (11)	C35—C36—C37—C32	0.3 (7)
C3—C4—C5—C6	0.9 (10)	C35—C36—C37—P3	179.8 (4)
C4—C5—C6—C1	−1.5 (9)	C26—P3—C37—C32	59.2 (5)
C2—C1—C6—C5	0.2 (9)	C38—P3—C37—C32	−51.8 (5)
P1—C1—C6—C5	178.9 (5)	Au3—P3—C37—C32	−177.0 (4)
C12—C7—C8—C9	−0.8 (9)	C26—P3—C37—C36	−120.2 (4)
C7—C8—C9—C10	−0.4 (9)	C38—P3—C37—C36	128.8 (4)
C8—C9—C10—C11	0.2 (9)	Au3—P3—C37—C36	3.5 (5)
C9—C10—C11—C12	1.0 (8)	C39—P4—C38—P3	−157.2 (3)
C8—C7—C12—C11	2.1 (8)	C50—P4—C38—P3	89.7 (3)
C8—C7—C12—P1	−177.5 (5)	Au4—P4—C38—P3	−33.2 (3)
C10—C11—C12—C7	−2.2 (7)	C26—P3—C38—P4	−172.3 (3)
C10—C11—C12—P1	177.4 (4)	C37—P3—C38—P4	−60.8 (3)
C13—P1—C12—C7	−151.7 (4)	Au3—P3—C38—P4	63.0 (3)
C1—P1—C12—C7	93.9 (4)	C50—P4—C39—C40	75.2 (5)
Au1—P1—C12—C7	−27.3 (5)	C38—P4—C39—C40	−34.9 (5)
C13—P1—C12—C11	28.7 (5)	Au4—P4—C39—C40	−159.9 (4)
C1—P1—C12—C11	−85.7 (4)	C50—P4—C39—C44	−103.2 (4)
Au1—P1—C12—C11	153.1 (4)	C38—P4—C39—C44	146.6 (4)
C25—P2—C13—P1	−176.5 (2)	Au4—P4—C39—C44	21.6 (5)
C14—P2—C13—P1	70.3 (3)	C44—C39—C40—C41	1.3 (8)
Au2—P2—C13—P1	−56.1 (3)	P4—C39—C40—C41	−177.1 (4)
C25—P2—C13—Au3	−64.4 (3)	C39—C40—C41—C42	−0.9 (8)
C14—P2—C13—Au3	−177.6 (2)	C40—C41—C42—C43	0.1 (9)
Au2—P2—C13—Au3	56.1 (2)	C41—C42—C43—C44	0.1 (9)
C1—P1—C13—P2	−70.8 (3)	C42—C43—C44—C39	0.3 (8)
C12—P1—C13—P2	178.6 (2)	C40—C39—C44—C43	−1.0 (8)
Au1—P1—C13—P2	56.1 (3)	P4—C39—C44—C43	177.4 (4)
C1—P1—C13—Au3	175.6 (2)	C50—C45—C46—C47	0.5 (8)
C12—P1—C13—Au3	64.9 (3)	C45—C46—C47—C48	1.1 (8)
Au1—P1—C13—Au3	−57.5 (2)	C46—C47—C48—C49	−0.9 (8)
Au4—Au3—C13—P2	111.11 (19)	C47—C48—C49—C50	−0.9 (8)
Au4—Au3—C13—P1	−128.78 (17)	C46—C45—C50—C49	−2.3 (7)
C13—P2—C14—C15	−136.6 (4)	C46—C45—C50—P4	172.7 (4)
C25—P2—C14—C15	110.6 (5)	C48—C49—C50—C45	2.5 (7)
Au2—P2—C14—C15	−9.2 (5)	C48—C49—C50—P4	−172.5 (4)
C13—P2—C14—C19	42.7 (5)	C39—P4—C50—C45	128.0 (4)
C25—P2—C14—C19	−70.1 (5)	C38—P4—C50—C45	−121.6 (4)
Au2—P2—C14—C19	170.1 (4)	Au4—P4—C50—C45	2.7 (4)
C19—C14—C15—C16	0.8 (8)	C39—P4—C50—C49	−57.1 (4)
P2—C14—C15—C16	−179.9 (4)	C38—P4—C50—C49	53.3 (4)
C14—C15—C16—C17	−1.1 (9)	Au4—P4—C50—C49	177.7 (3)
C15—C16—C17—C18	−0.4 (10)	C54—O1—C51—C52	−17.8 (8)
C16—C17—C18—C19	2.1 (10)	O1—C51—C52—C53	33.9 (8)
C15—C14—C19—C18	0.9 (8)	C51—C52—C53—C54	−38.2 (10)
P2—C14—C19—C18	−178.4 (4)	C51—O1—C54—C53	−6.9 (8)
C17—C18—C19—C14	−2.3 (9)	C52—C53—C54—O1	29.2 (10)
C25—C20—C21—C22	1.4 (8)	C58—O2—C55—C56	25.4 (10)
C20—C21—C22—C23	−3.0 (8)	O2—C55—C56—C57	−5.4 (11)
C21—C22—C23—C24	1.9 (8)	C55—C56—C57—C58	−15.1 (10)
C22—C23—C24—C25	0.9 (8)	C55—O2—C58—C57	−35.9 (8)
C21—C20—C25—C24	1.4 (7)	C56—C57—C58—O2	31.8 (9)
